# Antibacterial Action of Protein Fraction Isolated from *Rapana venosa* Hemolymph against *Escherichia coli* NBIMCC 8785

**DOI:** 10.3390/ph17010068

**Published:** 2024-01-03

**Authors:** Mihaela Kirilova, Yana Topalova, Lyudmila Velkova, Aleksandar Dolashki, Dimitar Kaynarov, Elmira Daskalova, Nellie Zheleva

**Affiliations:** 1 Faculty of Biology, Sofia University, 8 Dragan Tzankov blvd., 1164 Sofia, Bulgaria; ytopalova@sofia-uni.bg (Y.T.);; 2 Center of Competence “Clean Technologies for Sustainable Environment—Water, Waste, Energy for Circular Economy”, 1000 Sofia, Bulgaria; zhelevan@phys.uni-sofia.bg; 3 Institute of Organic Chemistry with Centre of Phytochemistry, Bulgarian Academy of Sciences, Acad. Georgi Bonchev str., bl. 9, 1113 Sofia, Bulgaria; adolashki@yahoo.com (A.D.); mitkokaynarov@abv.bg (D.K.)

**Keywords:** *Rapana venosa* hemolymph, *Escherichia coli* NBIMCC 8785, antimicrobial effect

## Abstract

Natural products and especially those from marine organisms are being intensively explored as an alternative to synthetic antibiotics. However, the exact mechanisms of their action are not yet well understood. The molecular masses of components in the hemolymph fraction with MW 50–100 kDa from *Rapana venosa* were determined using ImageQuant™ TL v8.2.0 software based on electrophoretic analysis. Mainly, three types of compounds with antibacterial potential were identified, namely proteins with MW at 50.230 kDa, 62.100 kDa and 93.088 kDa that were homologous to peroxidase-like protein, aplicyanin A and L-amino acid oxidase and functional units with MW 50 kDa from *R. venous* hemocyanin. Data for their antibacterial effect on *Escherichia coli* NBIMCC 8785 were obtained by CTC/DAPI-based fluorescent analysis (analysis based on the use of a functional fluorescence probe). The fluorescent analyses demonstrated that a 50% concentration of the fraction with MW 50–100 kDa was able to eliminate 99% of the live bacteria. The antimicrobial effect was detectable even at a 1% concentration of the active compounds. The bacteria in this case had reduced metabolic activity and a 24% decreased size. The fraction had superior action compared with another mollusc product—snail slime—which killed 60% of the *E. coli* NBIMCC 8785 cells at a 50% concentration and had no effect at a 1% concentration. The obtained results demonstrate the high potential of the fraction with MW 50–100 kDa from *R. venosa* to eliminate and suppress the development of *Escherichia coli* NBIMCC 8785 bacteria and could be applied as an appropriate component of therapeutics with the potential to replace antibiotics to avoid the development of antibiotic resistance.

## 1. Introduction

Antibiotic resistance is one of the major contemporary concerns related to human health [1]. It was estimated by the EU that 25,000 people lose their lives in the European Union each year because of infections with resistant bacterial strains, and 700,000 people in the world die yearly [1]. Up to 2050, the economic loss due to antibiotic resistance will be comparable to the 2008 economic crisis, according to the World Bank [2]. This is why one of the main priorities of the scientific investigations put forward by the World Health Organization and the EU is the discovery of new means for fighting bacterial infections [3,4]. The development of microbial resistance to conventional antibiotics and the emergence of new infectious diseases have led to the search for new potential sources of therapeutics. This is a global effort being conducted by investigators from various fields aiming to find new antibacterial compounds and encompasses the testing of synthetic as well as natural molecules [5,6,7]. In recent years, there has been a growing interest in marine natural products or marine-borne secondary metabolites [8]. However, studies on the antibacterial properties of the hemolymph of gastropods are very meager scarce [9].

Molluscs, which belong to the invertebrates, are a potentially rich source of bioactive molecules with antimicrobial properties, which has been proven in numerous studies [9,10,11,12]. The evolutionary survival of these organisms indicates that their immune system defenses are extremely efficient [12,13]. These organisms are known to lack the adaptive immune system found in vertebrates and rely solely on their innate immune system to resist invading pathogens [13]. The system of cells and biologically active compounds dissolved in their hemolymph or mucus acts synergistically against microorganisms [14,15,16]. Bioactive compounds such as AMPs and some proteins play important roles in the antimicrobial defense of molluscs [17]. While the healing properties of the mucus are well known and used in traditional medicine [18], the bioactivity of hemolymph against pathogens is yet to be elucidated.

Molluscan hemolymph is a body fluid that, like vertebrate blood, contains biomolecules with various functions, some of the most important being related to oxygen transport, antioxidant capacity and immune defense [12,19].

Some studies have focused on the entirety of the body fluid, showing its antibacterial features [19,20], and others show data on the differences in the hemolymph components regarding its antibacterial activity. A study by Amruthalakshmi and Yogamoorthi (2017) demonstrates the differences in hemocytes and hemocyte-free hemolymph against different pathogenic bacteria (*Klebsiella pneumonia*, *Staphylococcus aureus*, *Pseudomonas aeruginosa*, *Vibrio cholera*, and *Escherichia coli*) [9]. Tetreau et al. identified highly active proteins in hemocyte-free hemolymph from *Biomphalaria glabrata* [21]. Many studies have recognized the high potential of hemocyanins to participate in immune reactions [22,23,24]. Terwilliger highlighted their role as enzymes related to some immune reactions [25], while other studies show hemocyanin’s agglutinative activity [21,26] and its antibacterial activity [27]. Some authors even suggest that the antibacterial activity of molluscan hemolymph may be due to proteins and peptides derived from hemocyanins [27,28]. 

Recently, Krumova et al. reported for first time on the antifungal activity of three fractions of hemolymph from *Rapana venosa* against six fungal strains—*Fusarium oxysporum*, *Penicillium griseofulvum*, *Alternaria solani*, *Mucor hiemalis*, *Aspergillus niger*, *Botrytis cinerea*, and *Candida albicans* [29]. The results showed that only the fraction Rv 30–100 kDa manifested a broad spectrum with promising activity against all the tested strains, whereas the other two fractions, Rv 10 kDa (with MW below 10 kDa) and Rv 10–50 kDa, inhibited the growth of *F. oxysporum* and *F. oxysporum*, *P. griseofulvum* and *B. cinerea*, respectively, to some extent. Therefore, we focused our study on the bacterial activity of the fraction Rv 50–100 kDa.

The aim of the present study was to investigate the antibacterial effects and some of the mechanisms of inhibition of bacterial development due to the presence of biologically active substances in the fraction with MW 50–100 kDa from the hemolymph of *R. venosa* (Rv 50–100) in comparison with a peptide fraction with MW below 10 kDa from *C. aspersum* mucus.

## 2. Results

### 2.1. Analysis and Physicochemical Characteristics of the Isolated Fractions 

#### 2.1.1. Isolation of Fraction Rv 50–100 from the Hemolymph of Marine Snail *R. venosa*

**Figure 1:**
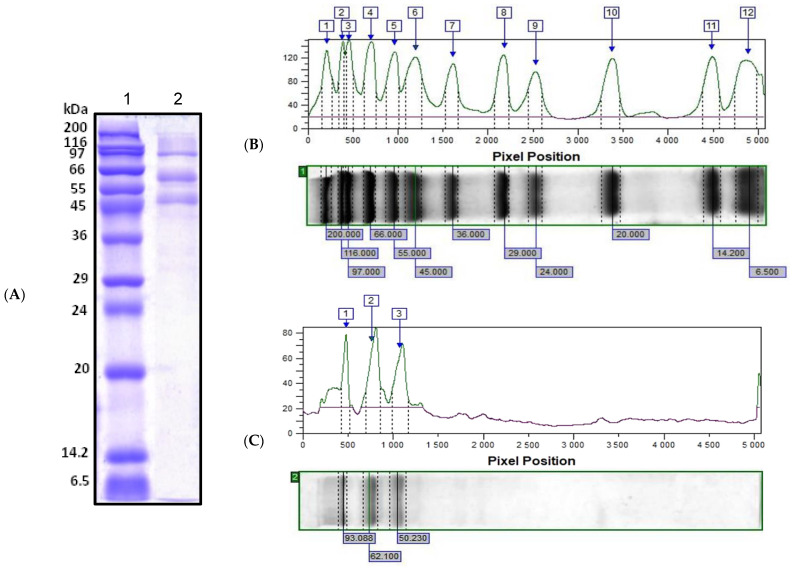
(**A**) Depiction of 12.0% SDS-PAGE analysis, visualized by staining with Coomassie G-250. Positions: (1) protein marker in the range 6.5–200 kDa (SigmaMarker^TM^, Sigma-Aldrich, Saint Louis, MO, USA); (2) fraction from *R. venosa* hemolymph with MW 50–100 kDa. (**B**) Electrophoretic profile of a standard protein molecular marker (electrophoretic Lane 1) analyzed by ImageQuant^TM^ TL. (**C**) Analysis of the electrophoretic profile of fraction Rv 50–100, electrophoretic Lane 1, using ImageQuant™ TL v8.2.0 software.

Hemocyanin, which is the main protein of the hemolymph of the marine snail *R. venos* (MW ~8000 kDa) [29,30], was removed by ultrafiltration under pressure using a Millipore membrane disc with MW 100 kDa, and the resulting fraction, with MW below 100 kDa, was additionally separated with a 50 kDa membrane. The obtained fraction, with MW between 50–100 kDa, contains mainly proteins with MWs of ~50 kDa, ~62 kDa and ~97 kDa, which was established by 12.0% one-dimensional denaturing (sodium dodecyl sulfate) polyacrylamide gel electrophoresis (1D-SDS-PAGE) (Figure 1). The obtained protein concentration of the fraction was 0.72 mg/mL.

After scanning with 12% SDS-PAGE and analysis using ImageQuant^TM^ TL v8.2.0 software, the exact MWs of the expressed proteins were determined at 93.088 kDa, 62.100 kDa and 50.230 kDa, respectively (Figure 1B,C). 

#### 2.1.2. Analyses of Fraction the with MW 50–100 kDa from the *R. venosa* Hemolymph 

In order to determine proteins in the fraction Rv 50–100 kDa, a search was performed in the database UniProt (https://www.uniprot.org, 10 November 2023) for proteins localized extracellularly in molluscs and gastropoda with MW corresponded with electrophoretic analysis (Figure 1).

Based on the conducted search, we hypothesized that the protein band at 93.088 kDa included peroxidase-like proteins identified in many Mollusca, such as in the hemolymph of *Lottia gigantea* (UniProt ID B3A0P3, with a theoretical MW 92.943 kDa); of *Margaritifera margaritifera* (UniProt ID H2A0M7 with MW 88.310 kDa); of *Mytilus coruscus* (UniProt ID A0A193DUA2 with MW 82.176 kDa); of *Mizuhopecten yessoensis* (UniProt ID A0A210Q736 with MW 89.489 kDa); of *Crassostrea virginica* (UniProt ID A0A8B8ATF7 and UniProt ID A0A8B8AEM7, MW 93.380 kDa and 91.888 kDa); of *Euprymna scolopes* (ID Q24925, MW 93.450 kDa); and of *Crassostrea virginica* (UniProt ID A0A8B8B7P3, MW 104.930 kDa). 

The next protein band at 62.100 kDa probably includes proteins with L-amino-acid oxidase (L-AAO) activity, found in the hemolymph of *Aplysia californica* (Uniprot ID: Q6IWZ0, MW 60.300 kDa), and Aplysianin A in *Aplysia kurodai* (Uniprot ID: Q17043, MW 62.376 kDa) and in *Physella acuta* (Uniprot ID: A0A8F1NMF6, MW 57.712). The protein band at 62.373 kDa probably includes proteins with L-amino-acid oxidase (L-AAO) activity, found in the hemolymph of *Aplysia californica* (Uniprot ID: Q6IWZ0, MW 60.300 kDa), and Aplysianin A in *Aplysia kurodai* (Uniprot ID: Q17043, MW 62.376 kDa) and in *Physella acuta* (Uniprot ID: A0A8F1NMF6, MW 57.712). 

The identified protein band at 50.230 kDa most likely included functional units (FUs) of *R. venosa* hemocyanin derived from endogenous proteolytic processes [30,31]. 

**Figure 2:**
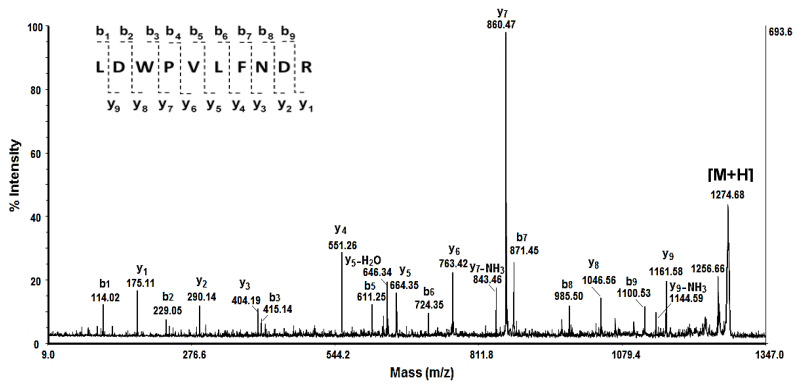
MS/MS spectrum of peptide [M + H]^+^ at *m*/*z* 1274.68 from the protein band at 61.106 kDa of the fraction MW 50–100 kDa of *R. venosa* hemolymph. The peptide amino acid sequence was determined manually following a series of b and y-fragment ions. After alignment of the identified amino acid sequence (LDWPVLFNDR) for peptide [M + H]^+^ at *m*/*z* 1274.68 with a database for extracellular proteins from gastropods by the Basic Local Alignment Search Tool (BLAST), we established a high homology (83%) with Aplysianin-A of *Aplysia kurodai.* In this way were determined the other proteins presented in Table 1 [31].

In order to confirm some of these proteins, the protein bands from 12% SDS-PAGE were excised from the gel and, after trypsin digestion, the extracted peptides were analyzed by mass spectrometry, as presented in study [31]. The amino acid sequences (AASs) for extracted peptides from each protein band were determined by de novo MS/MS analyses as described in [31], because Mascot search of the experimentally determined peptide masses as [M + H]^+^, after trypsin digestion, did not lead to satisfactory results. In Figure 2 is presented the MALDI-MS/MS spectrum of peptide [M + H]^+^ at *m*/*z* 1274.68 extracted from the protein band at 61.106 kDa. 

Alignment of the amino acid sequences identified for the peptides from protein bands at 50.230 kDa, 61.106 kDa and 93.088 kDa with known extracellular proteins in Gastropoda (taxid:6448) with the UniProtKB/SwissProt sequence database was performed with the blastp (protein–protein BLAST) algorithm. Most of the presented hits demonstrate identities above 60% and E-values between 1 × 10^−5^ and 1 (Supplementary Information 1 (SI 1)); only two of them have E-values above 1.0. It is known that the lower the E-value the more “significant” the match, suggesting a higher probability that the sequences share a common evolutionary origin. 

### 2.2. Antibacterial Activity of Fraction Rv 50–100 kDa against E. coli

The results from both cultivation and fluorescence analyses showed a strong inhibitory effect of the fraction with MW 50–100 kDa. This effect differs from the classical antibacterial response characterized by complete inhibition of the growth of the test microorganisms.

**Figure 3:**
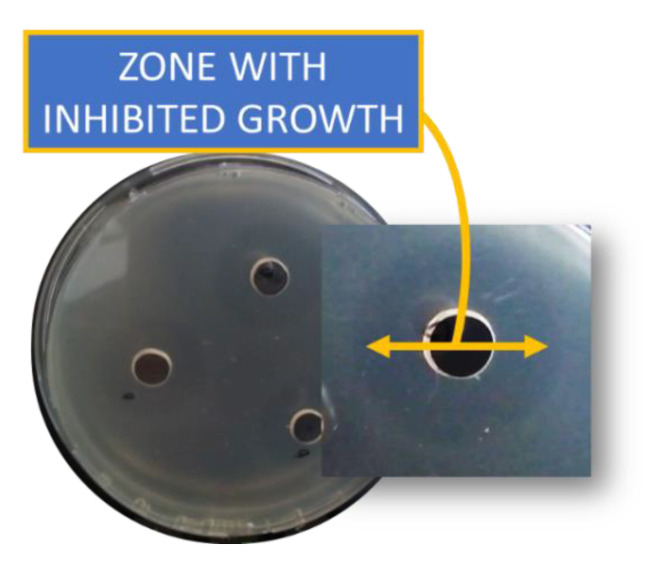
The well diffusion method for determination of the antibacterial activity of the fraction with MW 50–100 kDa. Deep inoculation of *E. coli* was applied.

Areas without growth of the test microorganisms were not registered when the protein fraction was inoculated onto the surface and within the depth of the medium for cultivation of *E. coli* NBIMCC 8785. At the same time, areas around the wells were found in which the growth of *E. coli* was inhibited. These areas can be clearly seen in the deep cultivation illustrated in Figure 3. This effect is likely attributed to the complexity of the investigated protein fraction. Our speculation was that the inhibitory effect occurs in two phases, influenced by proteins within the fraction with different mobility (transportation) in the nutrient media.

The measured antibacterial activity of undiluted *R. venosa* heolymph was exceptionally high compared with the antibacterial activity of snail mucus and the various peptide and protein fractions of snail mucus. This activity equates to 11,091.686 mm^2^/mgPr./µL of sample. In comparison, the highest activity of the most active fraction of snail mucus MW > 30 kDa was 894.00 mm^2^/mgPr./µL sample. This antibacterial activity is about 12 times higher. This gives reason to claim that when applying the hemolymph from *R. venosa* to treat bacteriostatic agents in the treatment of bacterial infections, solutions of the hemolymph can be used at different dilutions but achieve the same strong effect.

In order to establish the lowest effective hemolymph concentrations, our efforts were focused on a comparative study of the mechanisms of antibacterial action of hemolymph at different concentrations.

In order to obtain additional information about the inhibitory effect of the complex fraction with MW 50–100 kDa, a highly specific fluorescent method was applied, which allows the detection of the metabolic activity of pathogenic bacteria and changes in their morphology.

**Figure 4:**
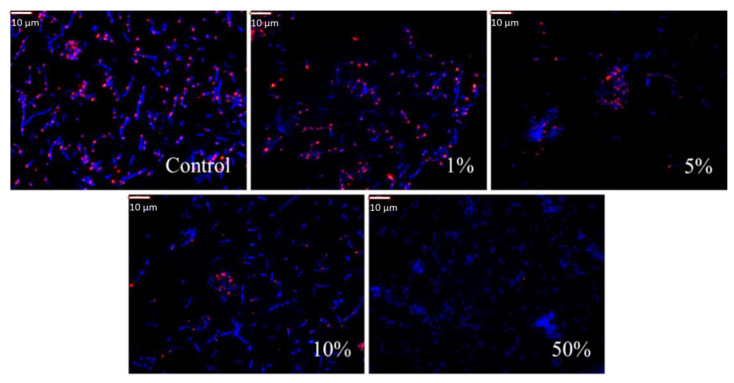
Fluorescence analysis for living and dead cells in *E. coli* bacteria after addition of 1%, 5%, 10% and 50% of the protein fraction with MW 50–100 kDa isolated from the hemolymph of *R. venosa* (all cells are colored blue with DAPI; only metabolically active cells are colored red with CTC). The images are with 1000× magnification.

**Figure 5:**
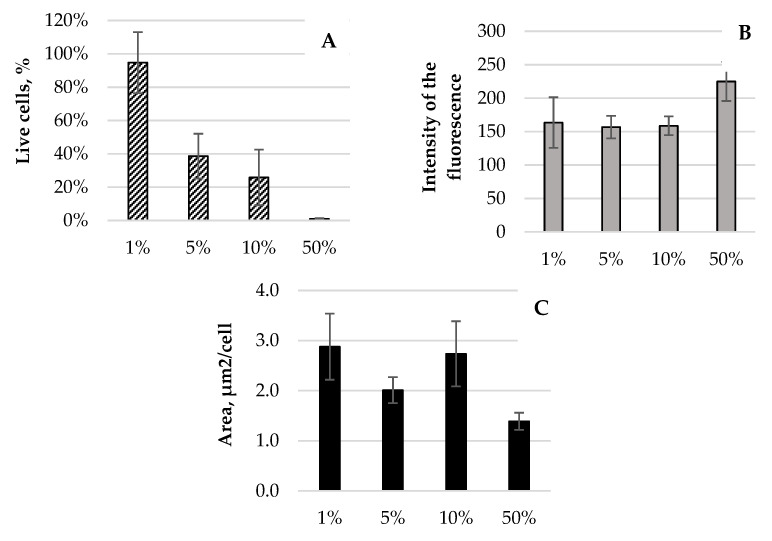
Digital analysis of images obtained from the fluorescent staining of *E. coli* samples with 1%, 5%, 10% and 50% of the protein fraction with MW 50–100 kDa from the hemolymph of *R. venosa*: (**A**) percentages of alive cells in the samples; (**B**) fluorescence intensity; (**C**) average areas of cells in the samples.

The results obtained showed a strong inhibitory effect of the protein fraction with MW 50–100 kDa on the reference strain of *E. coli* NBIMCC 8785. As depicted in the images in Figure 4, the number of living cells decreased with the increasing concentration of protein fraction. It was greatest when the incubation of the bacteria was with a fraction with concentration 1%. Figure 5A shows that the percentage of metabolically active cells in the samples was 95% (*p* = 0.001). When the concentration of the protein fraction from *R. venosa* increased to 5%, living bacterial cells decreased to only 39% after a 6 h incubation. Furthermore, with an increase in the concentration of the protein fraction from the hemolymph of the sea snail, the share of living cells decreased by another 13%; thus, only a quarter of the bacteria survived after 6 h incubation with the protein fraction of the snail.

At a high concentration (50%) of the protein fraction of *R. venosa*, almost all of the *E. coli* NBIMCC 8785 cells were destroyed after only one hour of incubation (Figure 5A). The proportion of metabolically active cells in the samples was 1%.

In addition to analyzing the share of active cells in the samples, a digital analysis of the fluorescent images was performed to obtain data on the intensity of cellular fluorescence (Figure 5B), as well as cellular average area (Figure 5C). These two parameters did not show a dose-dependent decrease because they reflect much more complex processes—the degree of metabolic activity at the individual cell level and the sizes of the cells. The latter can reflect the accumulation of biomass or effects on of the mechanisms of cell division. 

Fluorescence images can be used to obtain fluorescence intensity data and to detect changes in the metabolic activity of cells. Analysis of fluorescence intensity makes it possible not only to register the presence of alive cells but also to quantify their inhibition in the presence of the protein fraction from *R. vensa* hemolymph. This parameter provides valuable information for the practical application of *R. venosa*’s biologically active compounds, as inhibiting pathogenic bacteria would give a competitive advantage to the infected patient. This is a prerequisite to achieve the desired healing effect without completely destroying bacteria as a result of the presence of antimicrobial compounds. The data obtained show that the use of 1–10% of the fraction with MW 50–100 kDa from *R. venosa* leads to a decrease in intensity (Figure 5B). In all three variants, the decrease was approximately 25–28% (*p* = 0.02) compared with the control variant (Table 2). This is evidence that in addition to the effect of eliminating bacterial cells, the test fraction also had an inhibitory effect on those who survived the incubation period. From an applied point of view, it is important that this effect is registered even at the lowest concentration used. This would allow more rational use of valuable resources for its production.

When the highest concentration of protein fraction was applied, an increase in intensity was found (Table 2). However, it should be noted that the obtained data were based on the intensity of the few surviving bacterial cells. Evaluation of the morphological changes in *E. coli* cells through digital analysis of the fluorescent images was performed for two indicators—the average area of the imaged cells and their circularity. From the data shown in Table 2, it can be seen that at all four applied concentrations of the protein fraction there is a decrease in cell size.

The most pronounced decrease occurs at 5%, where the size of the cells in the samples was almost half the size in the control. At other concentrations of the protein fraction, a decrease of over 24% (*p* = 0.01) was also registered (Table 2). The effect was likely attributed to cell intoxication, which leads to reduced cell growth.

Circularity can provide information on morphological changes associated with cell elongation or rounding. The obtained data (Table 2) show that when applying 50% of the peptide fraction with MW below 10 kDa from *C. aspersum* mucus, the test microorganisms significantly reduced their circularity; i.e., not only were they reduced in size, but their cells had an altered elongated shape. No significant differences in cell shape were found at other concentrations of biologically active substances.

### 2.3. Comparison of the Antibacterial Activity of Fraction Rv 50–100 kDa and the Fraction with MW below 10 kDa from Cornu aspersum Mucus against of E. coli

To determine the prospects for application of the considered fraction with biologically active substances, its effects need to be compared with a similar biological product with already established properties. This is the peptide fraction with MW below 10 kDa from *Cornu aspersum* mucus. Our team already has significant data on it [12,32]. Based on these data, our hypothesis was that the antibacterial activity of the mucus fraction with MW < 10 kDa is due to a synergistic effect of cationic, anionic and neutral peptides with MWs of 0.800–2.500 kDa and peptides with a higher MW in the range of 3–10 kDa, some of them being glycopeptides and metabolites with low molecular weights.

**Figure 6:**
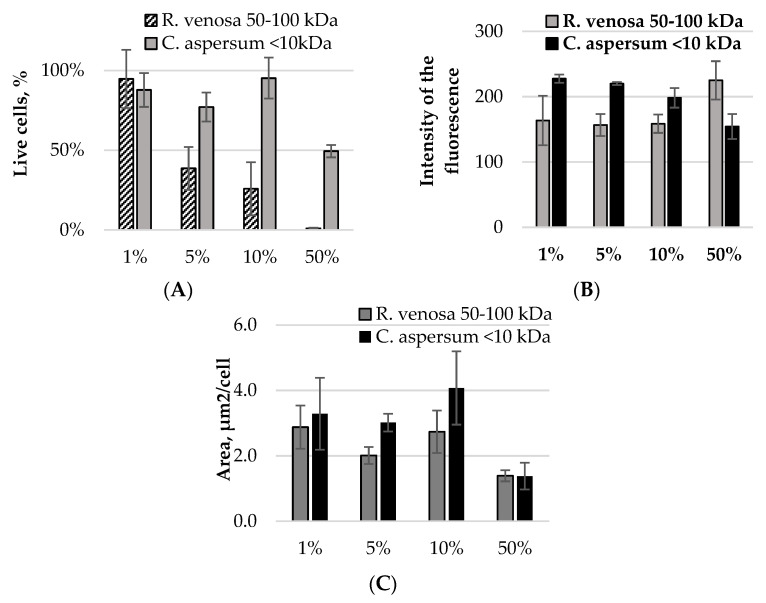
Digital analysis of images obtained from the fluorescent staining of *E. coli* samples with 1%, 5%, 10% and 50% of the fraction with MW 50–100 kDa from *R. venosa* and the fraction with MW below 10 kDa from *C. aspersum*: (**A**) percentages of living cells in the samples; (**B**) fluorescence intensity; (**C**) average areas of the cells in the sample.

The data in Table 2 show that this fraction also showed the most significant decrease in the proportion of living *E. coli* NBIMCC 8785 cells when the highest concentration of substances isolated from *C. aspersum* was applied compared with the control variant. However, this proportion (50%, Figure 6A) was 50 times lower than that using the fraction of *R. venosa* (1%, Figure 5A) (*p* = 0.006). The share of living bacterial cells was significantly lower when using 5% (2 times) and 10% (3.7 times) marine biologically active substances compared with terrestrial snails (Figure 6A). Using 1% of the fraction, a significant proportion of the test microorganisms remained active after 6 h of incubation (Figure 6A).

Fluorescence intensity analysis showed that at lower concentrations of active substances the effect of inhibition of bacterial metabolism was stronger in the fraction of *R. venosa* (Figure 6B) (*p* = 0.04). When using 1–10% of the active compounds, the decrease in fluorescence intensity was 25–28% compared with the control. When the substances isolated from *C. aspersum* were applied, no decrease in fluorescence intensity was found at 1% or 5%, while at 10% it was reduced by 9.5% compared with the control (Table 2).

In the presence of 50% of the fraction from *R. venosa*, an increase in the intensity of the few surviving cells was registered. In the variant with *C. aspersum*, the most significant decrease in *E. coli*’s metabolic activity was found (19%) at a 50% concentration. However, this reduction occurred when only half of the cells were alive compared with the control variant.

Morphological changes in *E. coli* cells after the action of both investigated peptide and protein fractions were associated with decreased cell size. This reached 34% compared with the control for both molecular fractions used at concentrations of 50% (Table 2). At concentrations of 1–10%, smaller cell sizes for the test microorganisms were recorded in the presence of proteins from the hemolymph of *R. venosa* (Figure 6C) (*p* = 0.02). The data confirm the results for fluorescence intensity being associated with a lower activity of bacterial metabolism in this experimental variant.

The data obtained show that the protein fraction from *R. venosa* has a much stronger antibacterial effect towards *E. coli* NBIMCC 8785 than the peptide fraction MW 10 kDa isolated from the mucus of *C. aspersum*. When the protein fraction from *Rapana* was used in low concentrations, a significantly greater reduction in the metabolic activity of the test microorganisms was registered. Moreover, at high concentrations and a one-hour exposure, almost complete destruction of bacterial cells was reached.

The described results highlight the promising potential of the fraction with MW 50–100 kDa from the hemolymph of *R. venosa* for use in clinical practice in *E. coli* infections.

**Figure 7:**
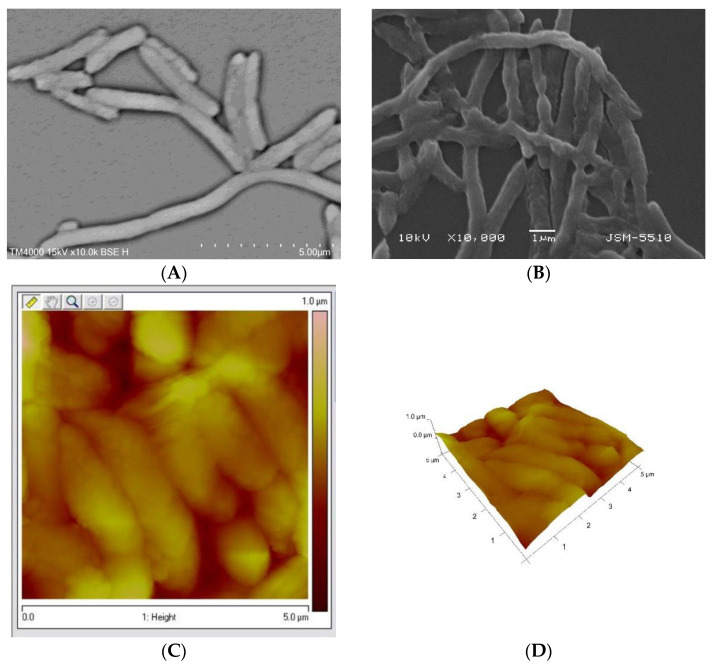
Illustration of the antibacterial damaging effect of a 10% solution of hemolymph from *Rapana venosa* against *E. coli* NBIMCC 8785. (**A**) control 18 h culture of *E. coli* NBIMCC 8785. (**B**) *E. coli* NBIMCC 8785 after 6 h exposure to rapana hemolymph—10% solution. The damage to the surface layers of the bacterial cells is clearly visible with SEM. (**C**) *E. coli* NBIMCC 8785 after 6 h exposure to rapana hemolymph—10% solution. The damage to the surface layers of the bacterial cells is clearly visible with AFM-2D. (**D**) *E. coli* NBIMCC 8785 after 6 h exposure to rapana hemolymph—10% solution. The damage to the surface layers of the bacterial cells is clearly visible with AFM-3D.

The results presented in Figure 7 confirm that concentrations of 5% and 10% of the fraction with MW 50–100 kDa from *R. venosa* hemolymph demonstrated a similarly pronounced antibacterial effect. Evaluation of this action based on a concentration/action comparison suggested that the 10% concentration was sufficiently antibacterially effective while being more economically and ecologically acceptable for therapeutic purposes. The fraction with MW 50–100 kDa from *R. venosa* hemolymph at this concentration causes severe damage to the surface layers of bacterial cells, which further leads to a bacteriostatic effect.

## 3. Discussion

The production of biologically active substances with antibacterial action from natural resources provides the prospect of obtaining new types of antimicrobial substances with reduced or even absent toxicity towards people but highly effective towards pathogens. Therefore, the efforts of many scientists are directed towards obtaining and characterizing biologically active substances of natural origin with a focus on the effectiveness and mechanisms of their impact on pathogens [5,33,34,35,36]. Molluscs, and gastropods in particular, are known to have high potential as sources of antimicrobial peptides and proteins. For example, the studies of Dolashki et al., 2020 [12], and Pitt et al., 2015 [37], demonstrated the presence of highly effective peptides in the mucus of terrestrial snails. Ulagesan and Kim [38] reviewed the antibacterial activity of a protein extract from seven species of terrestrial and freshwater snails. Sea snails are also a rich source of antimicrobial agents [39,40]. This is confirmed by the current study.

Several studies have shown that different proteins are present in the hemolymph of molluscs, such as different forms of hemocyanin, protease inhibitors, alpha-2-macroglobulin, a putative clotting protein, actin and many others [23]. Although, up to now, some extracellular proteins in *R. venosa* hemolymph have been identified, such as actin, some functional units of hemocyanin RvH, etc., as well as antimicrobial proline-rich peptides, the remaining proteins are still unknown [17,30,31,41]. 

Usually, analyses of peptide masses (measured by MALDI-Tof-MS after trypsin digestion) with Mascot Server peptide mass fingerprinting are widely used for protein identification when the protein of interest is in the database. However, in our case, this method did not lead to reliable identification, since the gene sequences are known for only some of the proteins in the hemolymph of *R. venosa*. Therefore, protein identifications were achieved based on the interpretation of de novo MS/MS analyses (Figure 2, Table 1).

The major proteins contained in the fraction Rv 50–100 kDa were determined based on the results of electrophoretic analysis and proteomic analysis on SDS-PAGE and compared with known extracellular proteins in Mollusca. We hypothesized that the protein with MW 93.088 kDa corresponds to a peroxidase-like protein. The presence of a peroxidase-like protein was also confirmed by the results of the proteomic analysis presented in Table 1. Similar proteins have long been associated with molluscan shell formation and probably act in the same way as the melanogenic peroxidase found in the ink gland of the cuttlefish *Sepia officinalis*, serving to cross-link proteins [42]. Moreover, peroxidases, one of the key antioxidant enzymes, are widely distributed in nature and catalyze the oxidation of various electron-donor substrates concomitant with the decomposition of H_2_O_2_. Some peroxidases exhibit antifungal activity [43,44]. Recently, in the fraction Rv 30–100 kDa, the presence of proteins with peroxidase activity was established, but not in the fraction Rv 10–50 kDa in another independent study [45]. 

The obtained results (Figure 2, Table 1) show that observed protein expression at 62.100 kDa corresponds to proteins with L-amino acid oxidase activity, which is also present in 18 more molluscan species [46,47]. These proteins play a variety of roles in the innate immune defenses of animals by catalyzing the oxidative deamination of L-amino acid substrate to alpha-keto acid, ammonia and hydrogen peroxide [47]. Moreover, in some gastropod species, more than one type of LAAO is often expressed [47]. Aplysianin A is the first protein from marine invertebrate animals with LAAO activity, which can specifically catalyze the oxidation of basic amino acids (L-arginine and L-lysine) [48]. The glycoprotein ‘achacin’ identified in the mucus of *A. fulica*, *Lissachatina fulica* and other snails also belongs to the family of amine oxidases [49,50]. Immunochemical analysis revealed that it preferentially binds to bacteria in the growth phase, which plays an important role in its antibacterial activity [50].

The presence of proteins with L-AAO activity in the protein band at 61.106 kDa in the Rv 50–100 kDa fraction may explain not only the observed antibacterial activity against *E. coli* but also the high antifungal activity of this fraction against six pathogenic strains [29], as well as recently established antitumor activity [31].

The functional units with MW 50.230 kDa identified in the hemolymph of *R. venosa* as a result of endogenous proteolytic processes may also be related to the observed antimicrobial properties of the Rv 50–100 fraction. A previous study reported antibacterial activity of hemocyanins from *H. aspersa* and *R. venosa* against *Staphylococcus aureus*, *Streptococcus epidermidis* and *E. coli* [51].

Summarizing these results, we hypothesize that proteins in the fraction 50–100 kDa from *R. venosa* hemolymph, which are homologous to peroxidase-like protein, aplicyanin A and L-amino acid oxidase (LAAO), and functional units with MW 50 kDa from RvH, may inhibit or kill *E. coli* cells by different mechanisms.

The data from microbiological analyses showed a strong antibacterial effect of the fraction with MW 50–100 kDa from the *R. venosa* hemolymph against *E. coli*. Nearly 60% of the bacterial cells were destroyed in 6 h using 5% of the active substances. The use of lower concentrations of biologically active substances aligns with a valuable trend that can meet modern demands for achieving strong antibacterial effects while saving resources by applying lower concentrations and smaller quantities of antibacterial agents. This would generally also exert lighter pressure on the environment as the antibacterial agents pass into wastewater after the treatment procedure. This minimalism with a preserved maximal effect against bacterial infections is modern and timely and should be pursued, especially when human and natural health are simultaneously pursued goals.

In present study, higher concentrations of the biologically active compounds were also investigated. When the concentration was increased to 50%, almost all microorganisms were destroyed in 60 min. Only individual active cells (1% of the cells) were found in the samples. They were registered to have higher metabolic intensity. This is normal, as they were left with a much larger amount of food resources to serve as a basis for metabolic transformation. With the actual application of such a concentration of the fraction, the human immune system would eliminate the remaining cells without difficulty.

The high efficiency in the elimination of *E. coli* was an indication that the compounds in the snail fraction with MW 50–100 kDa can be used as a means to overcome infections from this widespread pathogen. Murray et al. identify it as one of the six species of pathogenic microorganisms that pose a major threat to the emergence and spread of antibiotic resistance [52]. *E. coli* accounts for 23% of mortality directly caused by infections with AMR (antimicrobial resistance) microorganisms and 24% of mortality indirectly related to AMR in developed countries, with these microorganisms representing the largest share in most regions worldwide. Therefore, the WHO identifies them as bacteria with the highest priority in terms of antibiotic resistance. The WHO also highlights the critical importance of the development of new substances with antimicrobial activity affecting *E. coli*. The present study demonstrated the anti-*E. coli* activity of new natural substances that have a significant effect and that could act alone or in combination with conventional antibiotics to treat *E. coli* infections.

On the other hand, not only the elimination of bacteria but also the inhibition of surviving cells is important for the antibacterial effect. The results illustrated in Table 2 show that not only was the number of living pathogenic cells greatly reduced in the presence of the protein fraction from *R. venosa*, but the remaining living cells also had significantly lowered metabolic activity. Compared with the control, the decrease was approximately 27%. This was evidence of inhibition of the metabolic activity of *E. coli*.; i.e., the active substances in the studied fraction at concentrations of 1–10% caused the elimination of up to 72% of the bacteria. This was associated with serious morphological changes in pathogenic cells. Their size was up to 47% less than that of the bacteria in the control sample, which again confirmed the inhibition of their metabolism resulting in a decrease in cell size. These effects can be caused by various environmental stressors, including toxic substances [53]. The obtained results showed that the protein in the fraction with MW 50–100 kDa contained different types of proteins, so the biologically active ones were at even lower concentrations than the ones used as a complex fraction. A significant antibacterial effect was detected at the modest 5% concentration (Figure 5), making the possibility of low potency for the active compounds very unlikely.

It is of interest to compare the effects of the obtained fraction with new biologically active substances with a similar product, which has been well studied. That is why the fraction with MW below 10 kDa from *C. aspersum* was chosen. Our team has studied it in recent years, and it has been described in several publications and in a utility model [12,32]. Based on the determined amino acid sequences and physicochemical parameters, the analysis showed that the fraction with MW below 10 kDa contains cationic, as well as anionic and neutral, peptides. Most of the identified peptides belong to a new class of antimicrobial peptides rich in Gly/Leu that demonstrated antibacterial activity mainly against Gram-negative bacteria. The predicted antimicrobial activity determined by iAMPpred (online prediction server) showed various mucus peptides with high prognostic antibacterial, antifungal and antiviral activities.

The highest elimination effect on *E. coli* with the *C. aspersum* fraction was 60%, while the *R. venosa* fraction removed up to 99% of the bacterial cells in the samples (Table 2). At all concentrations, a reduction in the size of the bacteria was demonstrated, reaching half the size of the control microorganisms (47%). When the active compounds from *C. aspersum* were applied, the morphological change in the cells was lower at each of the concentrations, except for the highest. The mean effect of the decrease in the size of the bacteria was 15%, while, when the fraction of *R. venosa* was applied, it was twice as large (33%). The decrease in the metabolic activity of the test microorganisms when applying lower concentrations of the biologically active substances was also more pronounced for the fraction of *R. venosa* (average 27%). In the case where *C. aspersum* mucus compounds were applied, on average it was only 2%. The obtained results show that the protein fraction from *Rapana* with MW 50–100 kDa has an inhibitory effect against *E. coli*, which is superior to that of the peptide fraction with MW below 10 kDa, isolated from *C. aspersum*.

Most studies on molluscs, and in particular on snails, have identified substances with antimicrobial activity, such as peptides, defensins and others, that have a significantly lower molecular weight than 50 kDa [12,32,38]. On the other hand, the hemolymph of these animals contains large molecules of hemocyanin, which has known antibacterial properties [34,54]. Separate studies have demonstrated the production of peptides and proteins with an antimicrobial activity that is the result of the proteolysis of large molecules of hemolymph with a completely different action (histones and hemocyanin) [28,55]. It is likely that the used fraction contains similar or other unstudied proteins that have a strong inhibitory effect on *E. coli* and therefore open up great prospects for inclusion in preparations for therapy with antimicrobial action.

The study showed an extremely high antibacterial effect of a protein fraction of 50–100 kDa isolated from *R. venosa* against *E. coli* NBIMCC 8785.

By combining two well-known fluorescent dyes (CTC and DAPI), we performed an innovative and very sensitive analysis to estimate diverse antibacterial effects (share of live cells, metabolic activity and morphological changes in the bacteria). These changes were confirmed by SEM and AFM analyses while they were undetectable by standard microbiological methods. A CTC/DAPI-based fluorescence assay showed that the fraction with MW 50–100 kDa was able to eliminate 99% of live bacteria at a 50% concentration. Furthermore, even when treated with a concentration of 1% Rv 50–100 kDa, reduced metabolic activity and a 24% reduced size were observed.

Damage to the surface layers of *E. coli* NBIMCC 8785 bacterial cells was detected with SEM after treatment with 10% *R. venosa* hemolymph solution.

It is hypothesized that the observed promising antibacterial activity of a fraction with MW 50–100 kDa from the hemolymph of *R. venosa* is due to the synergistic action between three major types of proteins homologous to peroxidase-like protein, aplicyanin A and L-amino acid oxidase (LAAO) and functional units with MW ~50 kDa from RvH.

The data discussed so far illustrate the strong potential of the fraction with MW 50–100 kDa isolated from the hemolymph of *R. venosa* for application to treat infections caused by *E. coli*. These results can serve as a basis for the development of therapeutic purposes, avoiding the development of antibiotic resistance. At the same time, such antimicrobial peptides and proteins are environmentally friendly and keep the environment safe after their discharge in water and waste. 

## 4. Materials and Methods

### 4.1. Isolation of Protein/Peptide Fractions

The hemolymph was obtained from the marine snail *R. venosa* collected from its natural habitat in the Bulgarian Black Sea. The foot muscles were cut, filtrated and centrifuged for 30 min, at 4000× *g* and 4 °C, to remove rough particles and hemocytes. To avoid possible proteolysis of the hemolymph, 1 mM phenylmethylsulphonyl fluoride (PMSF) was added at its purification. The obtained hemolymph was divided into fractions by ultrafiltration, using membranes with pore sizes of 50 and 100 kDa (EMD Millipore Corporation, Billerica, MA, USA). Using these methods, fraction Rv 50–100, containing intact compounds with MW between 50–100 kDa, was obtained.

The crud mucus from *Cornu aspersum* snails grown on Bulgarian farms was obtained using a special patented technology without injuring any snails. The peptide fraction with MW below 10 kDa was obtained by ultrafiltration as described previously [12,32].

### 4.2. SDS-PAGE Electrophoresis

The protein fraction Rv 50–100 with antibacterial activity from *R. venose* hemolymph was analyzed using 12% sodium dodecyl sulfate-polyacrylamide gel electrophoresis (SDS-PAGE); equal volumes containing approximately 20 μg sample were dissolved in Laemmli buffer in the presence of dithiotheriotol (DDT) and boiled for 5 min at 100 °C, according to the Laemmli method with modifications [56]. The proteins were visualized by staining with Coomassie Brilliant Blue G-250. A molecular marker of standard proteins of 6.5–200.0 kDa (from Sigma-Aldrich) was used. The concentration of the protein fraction Rv 50–100 kDa was determined to be 0.72 mg/mL by Bradford assay, as described in [57].

### 4.3. Image Analysis of 12% SDS-PAGE with ImageQuant™ TL v8.2.0 Software

The obtained polyacrylamide gel (PAG) was captured on an Image Scanner III (GE Healthcare), and the image was opened with the ‘1D gel analysis’ utility of the Image Quant TL v8.2 software (GE Healthcare Bio-Sciences AB, Uppsala Sweden), which is highly automated software for image analysis. All bands were identified manually, including those in the standard protein marker, with a pen tool. To compensate the intensity of the image background, the background was modified with the “image rectangle” setting. Analysis of the molecular weight of each band was performed using the protein data standard SigmaMarker^TM^ (Sigma-Aldrich, Saint Louis, MO, USA), wide range, mol wt 6500–200,000 Da. Automatically, horizontal bands were drawn to the individual bands of the MW marker and calculated with the cubic curve spline. Based on the precalculated number of bands in the marker, the number of bands tested was determined [58].

### 4.4. Tryptic In-Gel Digestion and Peptide Extraction

Protease digestion was run according to the work of Rosenfeld et al. with a slight modification [59]. The target protein bands excised from the SDS-PAGE gels were washed twice with a 150 µL mixture of 50% acetonitrile (ACN) and 200 mM NH_4_HCO_3_ each for 20 min at 30 °C to decolorize, as described previously in [31]. The digestion of proteins in gel was carried out with porcine trypsin (Promega, Madison, WI, USA). After drying the decolorized gels in the speedvac concentrator, a volume of 10 μL digestion buffer (50 mM ammonium bicarbonate, pH 7.8, containing modified trypsin) was added to them, and the Eppendorf tubes were kept on ice for 45 min to allow the gel pieces to be completely soaked with the protease solution. Digestion was performed overnight at 37 °C, the supernatants were recovered, and the resulting peptides were extracted twice with 35 μL of 60% ACN/0.1% HCOOH. The extracts were pooled and dried in the speedvac concentrator. 

### 4.5. Mass Spectrometry Analysis

The peptides extracted from the gel were analyzed with mass spectrometry (MS- and MS/MS-analyses) on AutoflexTM III high-performance MALDI-TOF and TOF/TOF systems (Bruker Daltonics, Bremen, Germany), which use a 200 Hz frequency-tripled Nd–YAG laser operating at a wavelength of 355 nm. Analysis was carried out using α-cyano-4-hydroxycinnamic acid (CHCA) as a matrix. A total of 2.0 μL of the sample was mixed with 2.0 μL of matrix solution (7 mg/mL of CHCA) in 50% CN containing 0.1% TFA, and only 1.0 μL of the mixture was spotted on a stainless steel 192-well target plate. The samples were dried at room temperature (~20 °C) and subjected to mass analysis. A total of 3500 shots were acquired in MS mode, and a collision energy of 4200 was applied. The mass spectrometer was externally calibrated with a mixture of angiotensin I (1296.6848 Da), angiotensin II (1046.5418 Da), Glu 1—fibrinopeptide B (1569.65 Da), ACTH (1–17) B (1569.65 Da) and ACTH (18–39) (2465.1983 Da). The instrument was externally calibrated with fragments of Glu-fibrinopeptide B for MS/MS experiments. The amino acid sequences of the peptides were identified by precursor ion fragmentation using MALDI-MS/MS analysis. Database SwissProt and NCBI BLAST were performed with the amino acid sequences revealed by manual interpretation of the MS/MS spectra. The parameters for BLAST alignment were Swiss-Prot database sequences for gastropods (taxid:6558) with the organism defined as snail using the algorithm blastp (protein–protein BLAST). Other criteria included identities above 60% and E-values below 10 and 1 × 10^−6^; the molecular masses of the determined proteins were close to the molecular masses determined by ImageQuant^TM^ TL from 12% SDS-PAGE.

### 4.6. Experimental Design

The experiments in the present study focused on elucidating the antibacterial effects of the 50–100 kDa protein fraction isolated from *R. venosa* hemolymph. *Escherichia coli* NBIMCC 8785 were used as test microorganisms. The protein fraction, at concentrations of 1%, 5% and 10%, was added to the bacterial suspension in physiological saline. After 6 h of incubation, the samples were stained with 5-cyano-2,3-ditolyl tetrazolium chloride (CTC) to visualize the number and activity of bacteria. CTC is a colorless tetrazolium salt that is incorporated into the electron transport chains of bacteria and reduced to CTC formazan, which emits red fluorescence. The intensity of this fluorescence is proportional to the metabolic activity of the cells, which makes the technique suitable not only for the detection of living cells but also for obtaining information about their activity in specific conditions at the individual level. Staining with 4’,6-diamidino-2-phenylindole (DAPI) was also used because it gives information on the total number of cells and their morphology. In addition to the three concentrations of biologically active substances, analyses with a high protein concentration (50%) were performed, and a reduced exposure time of one hour was used for this incubation. Digital analysis of fluorescent images from all samples was performed to allow an objective quantitative assessment of the proportion of surviving cells, their activity, and morphological changes.

Therapeutic agents for infections based on antimicrobial peptide and protein fractions from the mucus of another species of snails—terrestrial snails—are widely used in traditional medicine and modern dermatology. The role of peptides with low molecular weight in this (below 10 kDa) has been proven, and they are used in various cosmetics. Therefore, it was of interest to compare the data on biologically active compounds from *R. venosa* to the results of a similar experiment with peptides with MW below 10 kDa isolated from *Cornu aspersum* mucus.

### 4.7. Test Microorganisms

A reference strain of *E. coli* NBIMCC 8785 was used as the test microorganism. The strain was delivered by the National Bank for Industrial Microorganisms and Cell Cultures, Bulgaria (NBIMCC), in a lyophilized state. Cells were revitalized and cultured according to NBIMCC instructions. An 18 h culture in nutrient media was prepared for the current experiments. Immediately before the work, the cells were washed with saline before centrifugation at 4000 rpm. The precipitate was resuspended to the original sample volume. Bacterial suspensions untreated with peptides, incubated in parallel with experimental variants with *R. venosa* and *C. aspersum* proteins, were used as control samples.

### 4.8. Protein/Peptide Fractions

Cultivation analysis. The antibacterial activity of the fraction with MW 50–100 kDa was studied by inoculation on the surface and in the depth of the solid nutrient agar medium. The method of well diffusion was used. Nutrient agar was used as the cultivation medium. The method has been described in detail [60]. The antibacterial effect was represented as the non-growth area around the wells, calculated as mm^2^/mgProten/µM sample.

### 4.9. Fluorescence Analysis

Samples from the experimental variants were stained with 5 mM CTC (Fluka, Charlotte, NC, USA) for 45 min and then with DAPI (Sigma Aldrich, Saint Louis, MO, USA) at a concentration of 1 µM/mL for 10 min. The fluorescent images were taken with a Leica DM6 B epifluorescence microscope with the same camera and LAS X Core software settings for all the samples.

The obtained images were digitally analyzed with DAIME 2.0 software [61]. The segmentation threshold was selected manually for each image. Information was obtained on (1) the percentage of living cells, calculated on the basis of the number of living cells (CTC) and the number of all cells (DAPI); (2) the fluorescence intensity of CTC, which is indicative of the metabolic activity of the cells; (3) the mean cell area (DAPI), which provides information about changes in the morphology of the test microorganisms; and (4) the average circularity of the cells, as this indicator can vary from 0 (line) to 1 (ideal circle). The data are indicative of the rounding or elongation of bacterial cells—an indication of destructive changes in them under the action of biologically active compounds from snails.

All analyses were performed in three independent replicates, and calculations for the two incubation time variants were performed based on the respective incubation time controls. The digital analysis was performed based on at least five combinations of CTC and DAPI images. The obtained data were processed with MS Excel, MS Office Professional 2019. Statistical analysis was performed using a *t*-test in Excel. Differences were considered statistically significant at the *p* < 0.05 level.

## 5. Conclusions

The discovery of new, more effective and selective, antimicrobial drugs of natural origin is one of the main trends in modern research. In the present study, we found that the fraction 50–100 kDa from *R. venosa* hemolymph has promising antibacterial activity against *E. coli* NBIMCC 8785, manifested by significant inhibition of metabolic activity, a reduction in cell viability of up to 99%, morphological changes, and death. We hypothesize that the observed promising antibacterial activity of the fraction with MW 50–100 kDa from *R. venosa* hemolymph is due to synergistic action between three main types of proteins, homologous to peroxidase-like protein, aplicyanin A and L-amino acid oxidase (LAAO), and functional units with MW ~50 kDa from RvH.

## Figures and Tables

**Table 1:** Amino acid sequences (AASs) of peptides, determined after analysis of their MS/MS spectra. Proteins were identified after comparing AASs with a database of protein sequences using the Basic Local Alignment Search Tool (BLAST) [31].

Band	AAS of Peptide	Mass exp. [M + H]^+^	Protein Name	UniProt ID	Identities
1	HGDDCCDMDMR	1297.41	Peroxidase-like protein 2 [*L. gigantea*]	B3A0P3	100%, E = 0.14
	DHGEPPYDDFR	1347.56	Peroxidase-like protein 2 [*L. gigantea*] Peroxidase-like protein 3 [*L. gigantea*]	B3A0P3 B3A0Q8	73%, E = 0.024 64%, E = 0.069
	LPGAFTGPTFNCIAR	1635.83	Peroxidase-like protein 3 [*L. gigantea*] Peroxidase-like protein 2 [L. *gigantea*]	B3A0Q8 B3A0P3	63%, E = 2 × 10^−4^ 63%, E = 0.017
2.1	MPAQPVAGLFDR	1301.58	Peroxidase-like protein 2 [*L. gigantea*]	B3A0P3	100%, E = 0.014
	LDWPVLFNDR	1274.70	Aplysianin-A [*Aplysia kurodai*] L-amino-acid oxidase LAAO [*Aplysia californica*]	Q17043 Q6IWZ0	83%, E = 0.67 70%, E = 0.16
	KLFWHMDWK	1290.67	L-amino-acid oxidase LAAO [*A. califonica*]	Q6IWZ0	63%, E = 1.6
	MFHFDELLDLPR	1532.81	L-amino-acid oxidase LAAO [*A. californica*] Aplysianin-A [*A. kurodai*]	Q6IWZ0 Q17043	86%, E = 0.24 86%, E = 0.35
	DYHFDELLDLMR	1566.76	Aplysianin-A [*A. kurodai*] L-amino-acid oxidase LAAO [*A. californica*]	Q17043 Q6IWZ0	55%, E = 0.059 55%, E = 0.12
	YDRWDVPEPEFVVLR	1919.98	Aplysianin-A [*A. kurodai*]	Q17043	63%, E = 9.6
2.2	TFAGFVLSGLGTSAR	1483.79	Hemocyanin type 2 unit-e; RvH2-e [*Rapana venosa*]; Hemocyanin 2, KLH-B [*Megathura crenulata*] Hemocyanin 1; KLH-A [*M. crenulata*] Hemocyanin type 2 unit a, RtH2-a [*R. venosa*]	P83040 Q10584 Q10583 P80960	85%, E = 2 × 10^−5^ 92%, E = 2 × 10^−5^ 85%, E = 3 × 10^−5^ 77%, E = 1 × 10^−4^
	EYRYYWDWQER	1693.78	Hemocyanin 1; Keyhole limpet hemocyanin A (KLH-A) [*M. crenulata*] Hemocyanin 2; KLH-B [*M. crenulata*]	Q10583 Q10584	83%, E = 0.024 83%, E = 0.024
3	GHKKRIRK	1022.68	Hemocyanin type 2 unit a, RtH2-a [*Rapana venosa*]	P80960	75%, E = 0.10
	IWATWQTLQK	1274.47	Hemocyanin 2 KLH-B [*M. crenulata*] Hemocyanin 1; KLH-A [*M. crenulata*] Hemocyanin 2-c chain, KLH2-c [*M. crenulata*] Hemocyanin type 2 unit a, RtH2-a [*R. venosa*]	Q10584 Q10583 P81732 P80960	80%, E = 2 × 10^−5^ 80%, E = 2 × 10^−5^ 78%, E = 3 × 10^−4^ 78%, E = 3 × 10^−4^
	DEVVPNPFVR	1171.61	Hemocyanin 1; KLH-A [*M. crenulata*] Hemocyanin type 2 unit a; RvH2-a [*R. venosa*] Hemocyanin 2; KLH-B [*M. crenulata*]	Q10583 P80960 Q10584	75%, E = 0.039 100%, E = 0.23 75%, E = 0.33
	VEITKALHKLGLR	1477.92	Hemocyanin 2, KLH-B [*M. crenulata*] Hemocyanin 2-c chain, KLH2-c [*M. crenulata*]	Q10584 P81732	64%, E = 0.002 64%, E = 0.20
	YHRQEHRRWWKD	1796.90	Hemocyanin 1; KLH-A [*M. crenulata*]	Q10583	83%, E = 0.58

**Table 2:** Percentage changes in the cellular intensity of fluorescence, circularity, area and living cells in the digital analysis compared with the control variants.

Peptide/Protein Fraction	Peptide/Protein Concentration	Intensity of Fluorescence, % (CTC)	Circularity, % (DAPI)	Area per Cell, % (DAPI)	Live Cells, %
*R. venosa* 50–100 kDa	1%	−25%	−2%	−24%	2.41%
	5%	−28%	5%	−47%	−58.29%
	10%	−28%	6%	−28%	−72.08%
	50%	17.65%	−17.65%	−34.15%	−99.20%
*C. aspersum* below 10 kDa	1%	3.93%	−9%	−13.58%	−5.07%
	5%	0.00%	−8%	−20.71%	−16.69%
	10%	−9.50%	−7%	7.18%	2.95%
	50%	−19.35%	−4%	−34.63%	−59.12%

Values were calculated on the basis of the *E. coli* control.

## Data Availability

Data is contained within the article and supplementary material.

## Supplementary Materials

The following supporting information can be downloaded at: https://www.mdpi.com/article/10.3390/ph17010068/s1, Supplementary Information 1 (SI1). An alignment of the amino acid sequences of some identified peptides from the protein bands at 50.230, 61.106 and 93.088 kDa of the fraction Rv 50–100 kDa with known extracellular proteins in Gastropoda (taxid:6448) in the database UniProtKB/SwissProt using the sequence algorithm blastp (protein–protein BLAST), filtered to match records with percent identity between 50 and 100 (https://blast.ncbi.nlm.nih.gov/Blast.cgi, 20 December 2023).
