# Supplementary material for: Antibacterial Action of Protein Fraction Isolated from Rapana venosa Hemolymph against Escherichia coli NBIMCC 8785
Source: Pharmaceuticals (Basel). 2024 Jan 3;17(1):68. doi: 10.3390/ph17010068 (PMC10821198; doi:10.3390/ph17010068)
Supplement: Supplementary file 1 [file pharmaceuticals-17-00068-s001.zip › pharmaceuticals-2761162-supplementary.pdf]

Supplementary Information 1 (SI 1):

SI 1. An alignment of amino acid sequences of some identified peptides from protein bands at 50.230 kDa, 61.106 kDa and 93.088 kDa of fraction Rv 50-100 kDa with known extracellular proteins in Gastropoda (taxid:6448) with database UniProtKB/SwissProt, sequences algorithm blastp (protein-protein BLAST), filtered to match records with identity between 50% and 100% (https://blast.ncbi.nlm.nih.gov/Blast.cgi).

Peptides from protein band at 93.088 kDa

1) HGDDCCDMDMR

> **RecName: Full=Peroxidase-like protein 2 [Lottia gigantea]**

Sequence ID: B3A0P3.1; Length: 884; Number of Matches: 1; Score = 21.0 bits (42), E value (Expect) **0.14**, Identities 5/5 (100%), Positives 5/5 (100%), Gaps 0/5(0%)

```
Query  2    GDDCC  6
            GDDCC
Sbjct  859  GDDCC  863
```

2) DHGEPPYDDFR

> **RecName: Full=Peroxidase-like protein 2 [Lottia gigantea]**

Sequence ID: B3A0P3.1; Length: 884; Number of Matches: 1; Score = 23.1 bits (47), E value (Expect **)** **0.024**, Identities 8/11 (73%), Positives 9/11 (81%), Gaps 0/11(0%)

```
Query  1    DHGEPPYDDFR  11
            DHG PPY+ FR
Sbjct  375  DHGVPPYNWFR  385
```

> **RecName: Full=Peroxidase-like protein 3 [Lottia gigantea]**

Sequence ID: B3A0Q8.1; Length: 294; Number of Matches: 2; Score = 21.8 bits(44) , E value (Expect) **0.069**, Identities 7/11(64%); Positives 8/11(72%); Gaps 0/11(0%)

```
Query  1    DHGEPPYDDFR  11
            DHG P Y+ FR
Sbjct  144  DHGLPSYNTFR  154
```

3) LPGAFTGPTFNCIAR

> RecName: Full=Peroxidase-like protein 3 [Lottia gigantea]

Sequence ID: B3A0Q8.1; Length: 294; Number of Matches: 1; Score = 29.9 bits(63); E value (Expect) **2e-04**, Identities 11/16(69%); Positives 11/16(68%); Gaps 1/16(6%)

```
Query  1    LPGAFTGPTFNC-IAR  15
            LPG   GPTF C IAR
Sbjct  196  LPGSLLGPTFSCLIAR  211
```

> RecName: Full=Peroxidase-like protein 2 [Lottia gigantea]

Sequence ID: B3A0P3.1; Length: 884; Number of Matches: 3; Score = 24.4 bits(50); E value (Expect) **0.017**, Identities 10/16(63%); Positives 10/16(62%); Gaps 1/16(6%)

```
Query  1    LPGAFTGPTFNCI-AR  15
            LPG   G TF CI AR
Sbjct  430  LPGSLLGETFSCIFAR  445
```

Peptides from protein band at 61.100 kDa

4) MPAQPVAGLFDR

> RecName: Full=Peroxidase-like protein 2 [Lottia gigantea]

Sequence ID: B3A0P3.1; Length: 884; Number of Matches: 1; Score = 24.0 bits(49); E value (Expect) **0.014**; Identities 7/7(100%); Positives 7/7(100%); Gaps 0/7(0%)

```
Query  2    PAQPVAG  8
            PAQPVAG
Sbjct  138  PAQPVAG  144
```

5) LDWPVLFNDR

> RecName: Full=L-amino-acid oxidase; Short=LAAO; Short=LAO; AltName: Full=Escapin; Flags: Precursor [Aplysia californica]

Sequence ID: Q6IWZ0.1; Length: 535; Number of Matches: 1; Score 20.6 bits(41); E value (Expect) **0.16**; Identities 7/10(70%); Positives 7/10(70%);Gaps0/10(0%)

```
Query  1    LDWPVLFNDR  10
            LDW  L NDR
Sbjct  335  LDWSQLRNDR  344
```

> RecName: Full=Aplysianin-A; Flags: Precursor [Aplysia kurodai]

Sequence ID: Q17043.1; Length: 556; Number of Matches: 1; Score 18.9 bits(37); E value (Expect) **0.67**; Identities 5/6(83%); Positives 5/6(83%);Gaps 0/6(0%)

```
Query  1    LDWPVL  6
            LDWP L
Sbjct  336  LDWPPL  341
```

6) KLFWHMDWK

RecName: Full=L-amino-acid oxidase; Short=LAAO; Short=LAO; AltName: Full=Escapin; Flags: Precursor [Aplysia californica]

Sequence ID: Q6IWZ0.1; Length: 535; Number of Matches: 1; Score 17.6 bits(34); E value (Expect) **1.6**; Identities 5/8(63%); Positives 6/8(75%);Gaps 1/8(12%)

```
Query  2    LFWHM-DW  8
            LF +M DW
Sbjct  384  LFSQMYDW  391
```

7) MFHFDELLDLPR

> RecName: Full=L-amino-acid oxidase; Short=LAAO; Short=LAO; AltName: Full=Escapin; Flags: Precursor [Aplysia californica]

Sequence ID: Q6IWZ0.1; Length: 535; Number of Matches: 2; Score 20.6 bits(41); E value (Expect) **0.24**; Identities 6/7(86%); Positives 6/7(85%); Gaps 0/7(0%)

```
Query  4    FDELLDL  10
            FDE LDL
Sbjct  200  FDEALDL  206
```

>RecName: Full=Aplysianin-A; Flags: Precursor [Aplysia kurodai]

Sequence ID: Q17043.1; Length: 556; Number of Matches: 2; Score 20.6 bits(41); E value (Expect) **0.24**; Identities 6/7(86%); Positives 6/7(85%); Gaps 0/7(0%)

```
Query  4    FDELLDL  10
            FDE LDL
Sbjct  201  FDEALDL  207
```

8) DYHFDELLDLMR

>RecName: Full=Aplysianin-A; Flags: Precursor [Aplysia kurodai]

Sequence ID: Q17043.1; Length: 556; Number of Matches: 3; Score 22.3 bits(45); E value (Expect) **0.059**; Identities 6/11(55%); Positives 8/11(72%); Gaps 0/11(0%)

```
Query  2    YHFDELLDLMR  12
            YHFD+ +  MR
Sbjct  489  YHFDDVMSTMR  499
```

>RecName: Full=L-amino-acid oxidase; Short=LAAO; Short=LAO; AltName: Full=Escapin; Flags: Precursor [Aplysia californica]
Sequence ID: Q6IWZ0.1; Length: 535; Number of Matches: 2; Score 21.4 bits(43); E value (Expect) **0.12**; Identities 6/11(55%); Positives 7/11(63%); Gaps 0/11(0%)

```
Query  2    YHFDELLDLMR  12
            YHFD+    MR
Sbjct  485  YHFDDVISTMR  495
```

9) YDRWDVPEPEFVVLR

>RecName: Full=Aplysianin-A; Flags: Precursor [Aplysia kurodai]
Sequence ID: Q17043.1; Length: 556; Number of Matches: 2; Score 16.8 bits(32); E value (Expect) **9.6**; Identities 5/8(63%); Positives 6/8(75%); Gaps 0/8(0%)

```
Query  2    DRWDVPEP  9
            DR  +PEP
Sbjct  458  DRSTIPEP  465
```

10) TFAGFVLSGIGTSAR

>RecName: Full=Hemocyanin type 2 unit e; AltName: Full=RtH2-e [Rapana venosa]
Sequence ID: P83040.1; Length: 413; Number of Matches: 1; Score 29.1 bits(61); E value(Expect)**2e-05**; Identities 11/13(85%); Positives 11/13(84%); Gaps 0/13(0%)

```
Query  2    FAGFVLSGIGTSA  14
            FAGF L GIGTSA
Sbjct  317  FAGFLLEGIGTSA  329
```

>RecName: Full=Hemocyanin 2; AltName: Full=Keyhole limpet hemocyanin B; Short=KLH-B; Flags: Precursor [Megathura crenulata]
Sequence ID: Q10584.2; Length: 3421; Number of Matches: 9; Score 32.5 bits(69); E value (Expect) **2e-05**; Identities 12/13(92%); Positives 12/13(92%); Gaps 0/13(0%)

```
Query  2     FAGFVLSGIGTSA  14
             FAGFVLSGI TSA
Sbjct  3225  FAGFVLSGIYTSA  3237
```

Sequence ID: Q10584.2; Length: 3421; Number of Matches: 9; Score 29.9 bits(63); E value (Expect) **4e-04**; Identities 12/14(86%); Positives = 12/14(85%); Gaps = 0/14(0%)

```
Query  1    TFAGFVLSGIGTSA  14
            TFAGF LS IGTSA
Sbjct  748  TFAGFFLSYIGTSA  761
```

Sequence ID: Q10584.2; Length: 3421; Number of Matches: 9; Score 28.6 bits(60); E value (Expect) **5e-04**; Identities 10/14(71%)Positives = 11/14(78%); Gaps = 0/14(0%)

```
Query  1    TFAGFVLSGIGTSA  14
            TFA F+LSG G SA
Sbjct  324  TFASFILSGFGGSA  337
```

>RecName: Full=Hemocyanin 1; AltName: Full=Keyhole limpet hemocyanin A; Short=KLH-A; Flags: Precursor [Megathura crenulata]
Sequence ID: Q10583.2; Length: 3414; Number of Matches: 9; Score 32.0 bits(68); E value (Expect) **3e-05**; Identities 11/13(85%); Positives 11/13(84%); Gaps 0/13(0%)

```
Query  2     FAGFVLSGIGTSA  14
             FAGFVLSGI T A
Sbjct  3217  FAGFVLSGIRTTA  3229
```

Sequence ID: Q10583.2; Length: 3414; Score 28.6 bits(60); E value (Expect) **5e-04**; Identities 10/13(77%); Positives 10/13(76%); Gaps 0/13(0%)

```
Query  2     FAGFVLSGIGTSA  14
             FAGF L G GTSA
Sbjct  1990  FAGFLLEGFGTSA  2002
```

Sequence ID: Q10583.2; Length: 3414; Score 28.2 bits(59); E value (Expect) **7e-04**; Identities 10/13(77%); Positives 10/13(76%); Gaps 0/13(0%)

```
Query  2     FAGFVLSGIGTSA  14
             FAGF L  IGTSA
Sbjct  1566  FAGFLLHNIGTSA  1578
```

>RecName: Full=Hemocyanin type 2 unit a; AltName: Full=Hemocyanin RHSS2 subunit; AltName: Full=Hemocyanin Rta; AltName: Full=Hemocyanin heavy structural subunit; AltName: Full=RtH2-a [Rapana venosa]
Sequence ID: P80960.2; Length: 407; Number of Matches: 2; Score 30.3 bits(64); E value (Expect) **1e-04**; Identities 10/13(77%); Positives 10/13(76%); Gaps 0/13(0%)

```
Query  2    FAGFVLSGIGTSA  14
            FAGF L GIG SA
Sbjct  300  FAGFLLRGIGSSA  312
```

11) EYRYYWDWQER

>RecName: Full=Hemocyanin 2; AltName: Full=Keyhole limpet hemocyanin B; Short=KLH-B; Flags: Precursor [Megathura crenulata]
Sequence ID: Q10584.2; Length: 3421; Number of Matches: 9; Score 23.1 bits(47); E value (Expect) **0.024**; Identities 5/6(83%); Positives = 5/6(83%); Gaps = 0/6(0%)

```
Query  5     YWDWQE  10
             YWDW E
Sbjct  1362  YWDWTE  1367
```

>RecName: Full=Hemocyanin 1; AltName: Full=Keyhole limpet hemocyanin A; Short=KLH-A; Flags: Precursor [Megathura crenulata]
Sequence ID: Q10583.2; Length: 3414; Number of Matches: 8; Score 23.1 bits(47); Expect **0.024**; Identities 5/6(83%); Positives 5/6(83%); Gaps =0/6(0%)

```
Query  5    YWDWQE  10
            YWDW E
Sbjct  107  YWDWTE  112
```

Peptides from protein band at 50.230 kDa

12) GHKKRIRK
>RecName: Full=Hemocyanin type 2 unit a; AltName: Full=Hemocyanin RHSS2 subunit; AltName: Full=Hemocyanin Rta; AltName: Full=Hemocyanin heavy structural subunit; AltName: Full=RtH2-a [Rapana venosa]
Sequence ID: P80960.2; Length: 407; Number of Matches: 1; Score 20.6 bits(41); E value (Expect) **0.10**; Identities 6/8(75%); Positives 6/8(75%); Gaps 0/8(0%)

```
Query  1  GHKKRIRK  8
          GH K IRK
Sbjct  3  GHSKKIRK  10
```

13) IWATWQTLQK

RecName: Full=Hemocyanin 2; AltName: Full=Keyhole limpet hemocyanin B; Short=KLH-B; Flags: Precursor [Megathura crenulata]

Sequence ID: Q10584.2; Length: 3421; Number of Matches: 8; Score 31.6 bits(67); E value (Expect) **2e-05**; Identities 8/10(80%); Positives 8/10(80%); Gaps 0/10(0%)

```
Query  1     IWATWQTLQK  10
             IWA WQ LQK
Sbjct  2728  IWAIWQALQK  2737
```

RecName: Full=Hemocyanin 1; AltName: Full=Keyhole limpet hemocyanin A; Short=KLH-A; Flags: Precursor [Megathura crenulata]
Sequence ID: Q10583.2Length: 3414Number of Matches: 8; Score 31.6 bits(67); E value (Expect) **2e-05**; Identities 8/10(80%); Positives 8/10(80%); Gaps 0/10(0%)

```
Query  1     IWATWQTLQK  10
             IWA WQ LQK
Sbjct  1068  IWAIWQALQK  1077
```

RecName: Full=Hemocyanin 2-c chain; AltName: Full=KLH2-c [Megathura crenulata]
Sequence ID: P81732.1; Length: 420Number of Matches: 1; Score 28.2 bits(59); E value (Expect) **3e-04**; Identities 7/9(78%); Positives 7/9(77%); Gaps 0/9(0%)

```
Query  2    WATWQTLQK  10
            WA WQ LQK
Sbjct  223  WAIWQALQK  231
```

RecName: Full=Hemocyanin type 2 unit a; AltName: Full=Hemocyanin RHSS2 subunit; AltName: Full=Hemocyanin Rta; AltName: Full=Hemocyanin heavy structural subunit; AltName: Full=RtH2-a [Rapana venosa]
Sequence ID: P80960.2; Length: 407; Number of Matches: 1; Score 26.1 bits(54); E value (Expect) **3e-04**; Identities 7/9(78%); Positives 7/9(77%); Gaps 0/9(0%)

```
Query  2    WATWQTLQK  10
            WA WQ LQK
Sbjct  209  WAIWQELQK  217
```

14) DEVVPNPFVR
>RecName: Full=Hemocyanin 1; AltName: Full=Keyhole limpet hemocyanin A; Short=KLH-A; Flags: Precursor [Megathura crenulata]
Sequence ID: Q10583.2Length: 3414Number of Matches: 2; Score 22.3 bits(45); E value (Expect) **0.039**; Identities 6/8(75%); Positives 6/8(75%), Gaps 0/8(0%)

```
Query  3     VVPNPFVR  10
             V PNPF R
Sbjct  2215  VMPNPFAR  2222
```

>RecName: Full=Hemocyanin type 2 unit a; AltName: Full=Hemocyanin RHSS2 subunit; AltName: Full=Hemocyanin Rta; AltName: Full=Hemocyanin heavy structural subunit; AltName: Full=RtH2-a [Rapana venosa]
Sequence ID: P80960.2; Length: 407; Number of Matches: 1; Score 20.2 bits(40); E value (Expect) **0.23**; Identities 5/5(100%);Positives 5/5(100%), Gaps 0/5(0%)

```
Query  6    NPFVR  10
            NPFVR
Sbjct  114  NPFVR  118
```

>RecName: Full=Hemocyanin 2; AltName: Full=Keyhole limpet hemocyanin B; Short=KLH-B; Flags: Precursor [Megathura crenulata]

Sequence ID: Q10584.2; Length: 3421; Number of Matches: 4; Score 19.7 bits(39); E value (Expect) **0.33**; Identities 6/8(75%); Positives 6/8(75%); Gaps 0/8(0%)

```
Query  1     DEVVPNPF  8
             D VV NPF
Sbjct  2218  DAVVNNPF  2225
```

15) VEITKALHKLGLR

>RecName: Full=Hemocyanin 2; AltName: Full=Keyhole limpet hemocyanin B; Short=KLH-B; Flags: Precursor [Megathura crenulata]

Sequence ID: Q10584.2; Length: 3421; Number of Matches: 9; Score 26.5 bits(55); E value (Expect) **0.002**; Identities 8/10(80%); Positives 9/10(90%); Gaps0/10 (0%)

```
Query  2     EITKALHKLG  11
             +IT ALHKLG
Sbjct  1626  DITSALHKLG  1635
```

>RecName: Full=Hemocyanin 2-c chain; AltName: Full=KLH2-c [Megathura crenulata]

Sequence ID: P81732.1; Length: 420; Number of Matches: 1; Score 21.0 bits(42); E value (Expect) **0.20**; Identities 7/11(64%); Positives 7/11(63%); Gaps 0/10 (0%)

```
Query  2    EITKALHKLGL  12
            EI  ALH L L
Sbjct  369  EISQALHDLDL  379
```

16) YHRQEHRRWWKD

RecName: Full=Hemocyanin 1; AltName: Full=Keyhole limpet hemocyanin A; Short=KLH-A; Flags: Precursor [Megathura crenulata]

Sequence ID: Q10583.2; Length: 3414; Number of Matches: 1; Score 19.3 bits(38); E value (Expect) **0.58**; Identities 5/6(83%); Positives 5/6(83%); Gaps 0/6(0%)

```
Query  3     RQEHRR  8
             RQEH R
Sbjct  1983  RQEHSR  1988
```
